# Difficulties Arising from Eruption of a Third Molar

**Published:** 1882-08

**Authors:** 


### DIFFICULTIES ARISING FROM ERUPTION OF A THIRD MOLAR.

The patient, a young man of nineteen, was in usual health; though of a somewhat scrofulous diathesis. When first seen his face was much swollen, and jaws rigid, from the eruption of a right inferior third molar. The mouth could be opened only far enough to admit liquid food.

This state of things continued five or six weeks, when suppuration began, and soon an external fistulous opening was formed near the anterior edge of the masseter muscle; this was soon followed by others, until, all together, they numbered nearly a dozen, some in the sterno-mastoid muscle, but most of them in the masseter, which seemed to be involved in all directions; one opening appearing at the outer angle of the eye. The last one which formed was behind the ear, just over the mastoid process. The patient suffered a good deal, and lost flesh though appetite was good most of the time.

When he first asked advice we cautioned him against applying a poultice, fearing an external opening. Not satisfied with this, he went to a physician, who told him to apply a poultice, which he did accordingly.

On talking with the physician afterwards in regard to poulticing, he said there was no effect either one way or the other, but it satisfied the patient to be "doing something," hence he advised the poultice.

Now, if this be true, why do our dental authorities speak against it so decidedly?

Does it, or not, have the effect of bringing the pus to the surface?

Might this trouble of external suppuration, and, a scarred face for life, have been avoided by not poulticing? or in *any* way? What course of treatment would have been better than merely *letting alone.* The jaws could not be separated far enough to allow of extracting the tooth in front of it, when first seen.

